# Level and factors associated with compliance to iron-folic acid supplementation among pregnant women in rural Soro district, Hadiya Zone, Ethiopia: cross-sectional study

**DOI:** 10.1186/s40795-023-00765-2

**Published:** 2023-09-19

**Authors:** Tegegn Tadesse Arficho

**Affiliations:** School of public health, Wachamo University, Hosanna, Ethiopia

**Keywords:** Compliance, Ethiopia, Folic acid, Hadiya Zone, Iron, Pregnant women

## Abstract

**Background:**

Despite the advantages of iron and folic acid supplementation, the compliance status among pregnant women for the supplements is very low in Ethiopia. However, the factors found to be associated with the compliance of iron and folic acid supplementation varies depending on geographical locations and socio-cultural characteristics within the country. Therefore, this study assessed the compliance to iron and folic acid supplements and its associated factors among pregnant women in the rural Soro district, Hadiya Zone, Southern Ethiopia.

**Methods:**

Cross-sectional study design was applied to conduct the study. The total sample size was 274. This study was conducted from June 10 up to 20, 2018. Women who live in rural Soro district at least for 6months and gave live birth 12 months prior to the survey were included in the study. The study subjects were selected by applying the simple random sampling method. Independent variables with p-value less than or equal to 0.25 during bivariate analysis were candidate for multivariable analysis. Finally, during multivariable analysis the independent variables with P-value less than 0.05 were declared as factors significantly associated with compliance to iron-folic acid supplementation during pregnancy.

**Results:**

Of the whole study participants only 51(18.8%) women had taken iron folic acid supplements for at least 90 days during their last pregnancy. Women who had frequent visits to health facilities for ante natal care were more likely to be compliant with iron-folic acid supplements than their counterparts [AOR(95%CI) = 4.50(1.18, 17.14)].

**Conclusion:**

In this study, the higher proportion of pregnant women did not take adequate dose of iron and folic acid tablets during their last pregnancy. Women who had a frequent visit to health facilities for antenatal care were more likely to be compliant for iron folic acid supplements than their counterparts. Every effort should be made in the community and health facilities by concerned bodies working in the maternal health area to mobilize pregnant women to take the antenatal care for at least four times to achieve the minimum dose of iron and folic acid supplements.

## Introduction

Around 41.8% of pregnant women in the world are anemic with the half of the burden is due to iron deficiency [1–3]. It is a wide spread public health problem which accounts 20% of maternal death worldwide. Anemia during pregnancy is highest in Africa with 61.3% of pregnant women are anemic especially, in Sub Saharan Africa [4]. Studies show that 22 to 56.8% of pregnant women are anemic in Ethiopia with higher proportion of women affected in rural areas [5–7]. It indicates anemia is a moderate to severe public health problem among this group in Ethiopia [4–5].

Physiological requirement for iron and folic acid is highest during pregnancy and intake from diets taken does not fulfill an individual’s requirement at that period. World Health Organization (WHO) recommends every pregnant woman to take a daily dose of 30-60 mg of iron and 400 μg of folic acid supplements starting from early months of pregnancy as integral part of ANC program. It is ideal to take 180 iron folic acid doses before giving birth to prevent iron and folate deficiencies and related consequences. In areas with a higher prevalence of anemia, it is recommended that supplementation continues for three months postpartum. Currently, many countries are working to achieve pregnant women to take 90 and more tablets of iron-folic acid (IFA) during their pregnancy [1, 3, 5, 8]. Moreover, in middle and low income countries iron and folic acid supplementation is very important intervention strategy formulated to prevent and reduce anemia during pregnancy, and to reduce pre-term birth, early neonatal death, low birth weight and birth defects among newborns related to iron and folic acid deficiencies [1–2].

Government of Ethiopia developed nutrition strategies and programs, and antenatal care (ANC) protocol that recommend routine provision of the iron and folic acid supplements for pregnant women. It is aimed at increasing proportion of pregnant women supplemented with IFA more than 50% [9–10]. However, 2016 Ethiopian demographic health survey (EDHS) report indicates that only 5% of pregnant women swallowed IFA supplements for at least 90 days during their pregnancy which opposes the WHO recommendation [5].

Several studies show that mother’s age, educational status, knowledge of anaemia and iron-folate supplements, history of anaemia and household wealth index are associated with compliance to iron and folic acid supplements in Ethiopia [11–13]. Besides, fear of side effects, forgetting to take medications at regular time, irregular ANC follows and shortages related to supply side were factors associated with incompliance to iron-folic acid supplement utilization [14–16]. However, the factors found to be associated with compliance to IFA supplementation differs depending on geographical locations and socio-cultural characteristics. Moreover, many studies were conducted to assess the adherence to supplements based on the pill count per week by taking a short period which might not indicate the overall doses of the IFA supplements taken during the whole pregnancy of the women. A few community based studies were conducted to assess the compliance to IFA supplements based on national protocol that recommends the supplements to be taken for at least 90 days pregnancy. Identifying the prevalence of the compliance and its associated factors based on the rural context is very important to support the maternal nutrition and health interventions being done to increase the compliance of IFA among pregnant women. Therefore, this study assessed the prevalence of the compliance to iron-folic acid supplements and associated factors among women during pregnancy in rural Soro district, Hadiya Zone, Southern Ethiopia.

## Methods

### Study setting

Soro district is one of the ten districts and two urban administrations in Hadiya zone. It is located in 262 km from Addis Ababa and 32 km from zonal capital, Hossana. The total population of Sorro district is 268,281 and female accounts 134,673(50.2%) as reported by the district’s finance and economic development office in 2017G.C. The total number of households at the district estimated to be 29,126 [17]. The district has 10 health centers and 49 health posts. The health facilities provide free ante natal care, delivery and post natal care for service seekers from their catchment areas. Iron and folic acid supplements are one of the services to be provided free of charge for pregnant women in public health facilities found in the study area. This study was conducted from June 10 up to 20, 2018.

### Study design

Community- based cross-sectional study design was employed to conduct the study.

### Inclusion and exclusion criteria

All mothers who live in the study area and gave birth within 12 months since giving birth for their last or index child were included in the study. However, those mothers who were severely sick and unable to respond due to illness were excluded from the study.

### Source and study population

The source population was all mothers with children less than one year and living in the rural Soro district. The study population of this study was all mothers those were randomly selected for the study.

### Sample size determination

To estimate the sample size for compliance of IFA supplementation among women single proportion formula was used by using the following assumptions: Anticipated Proportion of mothers compliant of IFA supplementation is 20.4% [12], standard normal value at 95% confidence level(1.96) and margin of error 5%. By considering non-response rate of 10%, the final sample size was 274.

### Sampling techniques and procedures

Twelve rural *kebeles* (smallest administrative units) were selected randomly by using the lottery method out of 46 rural *kebeles* found in the Sorro district. The list of households having women with children less than one year was registered by using health development armies from each selected *kebeles*. Then the sample size was allocated proportionally for each *kebele*. After preparing the sampling frame simple random sampling method was applied to collect data from the index mother.

### Operational definition

A mother was categorized as compliant to IFA supplements if she had taken at least 90 doses of iron-folic acid supplements during her last pregnancy [18].

Knowledge about anemia was assessed by asking questions about causes, symptoms, prevention and treatment of anemia. The level of the knowledge according to IFA was assessed by asking questions about uses of IFA tablets, duration of adequate intake, time of starting supplements and side effects. Correct answer scored one point and incorrect answer scored zero. Those who had correctly answered more than 80% of anemia and iron-folic acid knowledge assessing questions were decided as having high knowledge about anemia and IFA. Additionally, those mothers who had correctly responded 60–80% and less than 60% of knowledge assessing questions were categorized as having medium and low knowledge about anemia and the IFA, respectively [12].

### Data collection procedures

The data were collected by using pretested a structured questionnaire which was prepared by English language and translated into Hadiyissa (local language) and back translated to English by fluent speakers of the languages. Intensive training was given for supervisors and data collectors on the purpose of study, how to handle questionnaires, how to conduct data collection and on ethical consideration. Then data were collected using twelve trained female enumerators who were fluent in local language and with prior experience of participation in data collection. Four BSc health professionals were participated on supervision of the data collection process. 5% of the questionnaires were pre tested in 1st Salfe kebele before actual data collection period and the local name of the iron-folic acid was added (they usually call it xiggi eddo Kashar qaracho). Strict supervision was done by supervisors and the overall quality of the data collection was monitored by investigators of the study. The enumerators submitted the data they collected to the supervisors on daily basis. The data were checked for completeness, consistency and any error was being corrected immediately.

### Study variables

The dependent variable was compliance to Iron and folic FA supplements.

The independent variables were:

Socio-demographic and economic characteristics, maternal health service related characteristics, obstetric characteristics and knowledge about IFA supplementation and anemia.

### Data processing, analysis and presentation

After the field work, the data were checked for completeness and consistence before data entry and cleaning. Then, the data were entered on Epi info version 3.5.4 and exported to SPSS for windows version 16 (SPSS Inc. version 16, Chicago, Illinois) to do the analysis. Descriptive summary was presented by using frequencies, proportions, means and tables. Household wealth index was assessed based on durable asset ownership (1 = yes and 0 = no) and analyzed by using principal component analysis (PCA). PCA was applied by checking the Kaiser–Meyer–Olkin (KMO) test(> 0.5), Bartlett’s test of sphericity(< 0.05), anti-image, communality and presence of a variable with complex structure. Both bivariate and multivariable logistic regression analysis were used to assess the association of independent variables with outcome variable. Compliance of IFA supplements was coded as “1” while noncompliance of IFA supplements was coded as “0” for running logistic regression analysis. Odds ratio with 95% confidence interval was used to assess the strength of the association. In the binary logistic regression model, independent variables with p-value less than or equal to 0.25 during bivariate analysis were a candidate for multivariable analysis. Finally, during multivariable analysis an independent variable with P-value less than 0.05 was declared as factor significantly associated with compliance to iron-folic acid supplementation during pregnancy. The model fitness was checked by Hosmer-Lemishow test of fitness being non-significant (> 0.05).

## Result

### Socio demographic characteristics of the respondents

From the total of 274 mothers who were eligible for the study, 271 were participated in the study making the response rate of 98.9%. The mean age of the respondents was 26.83(± 5.25) years with the minimum age of 15 and the maximum age 43. More than half of the mothers (55.7%) were within an age of 25–34 years. According to the marital status, most of the respondents (95.6%) were married and living with their husbands. About 90% of the respondents were protestant religion followers. According to the ethnicity most of the respondents were Hadiya (90.4%). About two-thirds (75.3%) of the participants were housewives. According to the educational status, about 40% of the respondents didn’t attend any education (Table 1).

**Table 1:** The Socio demographic characteristics of mothers in Sorro district, Hadiya Zone, South Ethiopia, 2018 (n = 271)

Variable	Category	n	%
Age of mother (years)	15–24	91	33.6
	25–34	151	55.7
	≥ 35	29	10.7
Religion of mother	Protestant	245	90.4
	Orthodox	13	4.8
	Catholic	12	4.4
	Muslim	1	0.4
Mother’s ethnicity	Hadiya	245	90.4
	Kambata	13	4.8
	Tambaro	13	4.8
Occupation of mother	Housewife	204	75.3
	Merchant	44	16.2
	Farmer	11	4.1
	Other*	12	4.4
Mother’s educational Status	Illiterate	107	39.5
	Read and write only	29	10.7
	primary	113	41.7
	Secondary and above	22	8.1
Wealth index	poor	132	48.7
	middle	46	17.0
	rich	93	34.3

* daily laborer, hand craft, government worker

### Maternal health service related and obstetric characteristics

More than two thirds of respondents (76.8%) had history of ANC visit for one and more times during their last pregnancy. About 34% of the pregnant women did not receive any iron and folic acid supplements from the public health facilities or bought from private clinics or drug stores during their pregnancy. More than half of the study subjects (66.4%) reported as they had consumed at least one tablet of IFA during their last pregnancy with median number of 45 supplements which ranges from 2 up to 140 tablets. The mean month of pregnancy to consume the first IFA supplement was 5.98(± 1.82). From the whole study participants only 18.8% (95%CI: 14.8, 22.8) were found to be compliant (swallowed for at least 90 supplements) to IFA supplementation during their last pregnancy.

Fear of the side effects related to the supplement intake (72.6%), negligence (poor attention on the importance of the supplements) (44.2%) and forgetfulness (35.8%) were the main reasons for not consuming the given IFA supplements daily among respondents. According to the knowledge of the women, 90% and 61.6% of women had higher knowledge about anemia and iron folic acid supplementation, respectively (Table 2).

**Table 2:** Maternal health service and obstetric related characteristics in Sorro district, Hadiya Zone, South Ethiopia (n = 271)

Variable	Category	N	%
Nearest health facility	Health post	181	66.8
	Health center	90	33.2
Number of children currently	1 up to 3 children	115	42.4
	4 up to 6 children	118	43.5
	7 and above	38	14.0
Birth order	first	31	11.4
	second	50	18.5
	Third and above	190	70.1
ANC received	yes	208	76.8
	no	63	23.2
Number of ANC	Once	9	3.3
	2–3 times	115	42.4
	4 and above	72	26.6
	Don’t know	12	4.4
IFA given	yes	180	66.4
	no	91	33.6
Compliant of IFA	yes	51	18.8
	no	220	81.2
IFA by categories	1–59 tablets	100	36.9
	60–89 tablets	29	10.7
	90 and above	51	18.8
Knowledge of anemia	Low knowledge	9	3.3
	Medium knowledge	15	6.6
	High knowledge	244	90.0
Knowledge of IFA	Low knowledge	86	31.7
	Medium knowledge	18	6.6
	High knowledge	167	61.6

### Factors associated with compliance to iron-folic acid supplements among pregnant women

Bivariate analysis revealed that mother’s educational being primary level, having ANC visit during pregnancy four and above times, household ownership of radio, knowledge about iron-folic acid supplements being higher and the number of children being 7 and above were factors associated with compliance of IFA supplementation. However, during multivariable logistic regression analysis having ANC visit 4 and above times during pregnancy was found to be significantly associated with optimal iron folic acid supplement intake (at least 90 supplements) among pregnancy women. The odds of taking iron-folic acid supplements among pregnant women those who had ANC visit 4 and above times were 4.5 times higher than their counterparts who had no ANC visit [AOR(95%CI) = 4.50(1.18, 17.14)](Table 3).

**Table 3:** Factors associated with adherence of IFA among pregnant women in Soro district, Hadiya Zone, South Ethiopia, 2018 (n = 271)

Variable			Compliant of IFA		COR(95%CI)	AOR(95%CI)
			yes	no		
Maternal education						
No education Primary Secondary and above			18(13.2) 28(24.8) 5(22.7)	118(86.8) 85(75.2) 17(77.3)	1 2.16(1.12,4.15) 1.93(0.63,5.87)	1 1.92(0.93, 3.95) 1.71(0.51, 5.73)
Antenatal care						
No ANC 1–3 times 4 and above ANC Don’t know			3(4.8) 27(21.8) 19(26.4) 2(16.7)	60(95.2) 97(78.2) 53(73.6) 10(83.3)	1 5.57(1.62,19.51) 7.17(2.01,25.60) 4(0.60,27.02)	1 3.79(1.04,13.81) 4.50(1.18, 17.14)* 4.29(0.59, 31.12)
Have radio in the household	yes		28(15.8)	149(84.2)	1.72(0.93,3.20)	1.21(0.62, 2.38)
	no		23(24.5)	71(75.5)	1	1
Number of children currently	1–3		19(16.5)	96(83.5)	1	1
	4–6		21(17.8)	97(82.2)	1.09(0.55,2.16)	1.18(0.56, 2.47)
	Above 7		11(28.9)	27(71.1)	2.06(0.87,4.85)	2.45(0.94, 6.38)
Knowledge about iron and folic acid supplements		high	38(22.8)	129(77.2)	2.08 (0.97,4.16)	1.56(0.71, 3.45)
		low	11(12.8)	75(87.2)	1	1

*p-value < 0.05

## Discussion

This study was aimed on assessing the compliance to iron and folic acid supplementation and its associated factors during pregnancy. The overall prevalence of the compliance to IFA is 18.8%(95%CI: 14.8, 22.8). Having antenatal care visit during pregnancy is found to be significantly associated with compliance to IFA supplementation.

In this study, the prevalence of compliance to IFA was 18.8%(95%CI: 14.8, 22.8) among mothers during their last pregnancy. The finding is similar with the previous studies conducted in Pakistan 16.9%, Kenya (18.3%) and Northern Ethiopia (20.4%) [12, 19–20]. However, the finding of this study is higher than that was reported in Ethiopian demographic health survey(EDHS) 2016 at which only 5% of the pregnant women took 90 and above tablets of IFA during their pregnancies [5]. In contrary to this, the status of adherence to IFA in this study is lower than the reports from studies conducted in Senegal (51%), Nepal (23%), India (23.8%) and Indonesia (34%) [18, 21–22]. This might be due to the differences in the level of the maternal care provision offered during pregnancy in different contexts. The difference in the reported prevalence of IFA from the Ethiopian demographic health survey’s finding might be due to the difference in characteristics from the mothers participated in the EDHS in the whole country and the Ethiopian EDHS uses the last five years as a reference to probe for intake of the supplements which might be more exposed for the recall bias.

The respondents who had antenatal care visit during their last pregnancy were more likely to be compliant to IFA consumption than their counterparts who had no antenatal care visit. However, the higher frequency of the antenatal care follow up during pregnancy is found to be associated with taking iron and folic acid supplements for at least 90 days in this study. The odds of taking iron-folic acid supplements among pregnant women those who had ANC visit 4 and above times were 4.5 times higher than their counterparts who had no ANC visit. Mothers who had ANC visit 1 up to 3 times during their last pregnancy had 3.79 times higher odds of swallowing iron and folic acid tablets than those who had no ANC visit. The finding implies that antenatal care service utilization particularly an increased frequency of the visit is an essential way to make pregnant mothers to utilize more doses of the IFA supplements. Iron and folic acid is being given as the integral part of ANC with free of charge in Ethiopia. The Ethiopian antenatal care guideline recommends daily oral iron and folic acid supplementation (60 mg elemental iron and 0.4 mg folic acid) for all pregnant women at least 90 tabs to the maximum 180 tabs. ANC service is the only channel for providing free iron and folic acid supplements for pregnant women in the country. Lack of access to get the IFA supplements through optional ways like home to home distribution might be a reason for mothers with no ANC follow up to be none compliant of supplementation than their counterparts. The finding is in line with the findings from studies done in India, Kenya and Sudan (19–20, 23) where the studies revealed that antenatal care service visit was associated compliance to iron and folic acid supplementation during pregnancy.

Another studies show that age of the mother [11, 13, 22], maternal educational status [11, 13], knowledge about anaemia and iron folate tablets [11, 19] were factors associated with consumption of IFA for 90 + days during pregnancy. However, these factors were not found to be significantly associated with intake of the supplements for the minimum recommend days in this study. This might due to the setup based differences in socio-demographic characteristics and health service delivery related to nutrition education and counseling for pregnant mothers.

This study has its own limitations. The study didn’t consider the design effect during the sample size calculation and the finding might be prone for the sampling error due to using a small sample size. The result of this study could be prone to recall bias because of error in recalling the past history related to their last pregnancy. However, mothers who gave birth within one year prior to the interview date were used to reduce the recall bias among the study subjects. Another weakness of this study is the nature of the study which can not indicate the cause and effect relationship between the exposure and outcome.

### Conclusion and recommendations

In this study, the higher proportion of pregnant women did not take adequate doses of iron and folic acid supplements during their last pregnancy. Having attendance for antenatal care service especially an increased follow-up visit is significantly associated with compliance to iron and folic acid supplementation based on the minimum dose recommendation by WHO for pregnant women. Every effort should be done in the rural communities to encourage all pregnant women to join the ANC services in their nearby health facilities. Besides, primary health workers and other concerned bodies should encourage and educate pregnant women to frequently visit when pregnant women are once registered and start the ANC service. This study did not study the factors affecting compliance to iron and folic acid supplementation related to the supply side. Studies in the future should consider issues related to the supply of IFA for primary health care units especially in rural setups.

## Data Availability

The dataset generated and/or analysed during the current study are available in the repository (https://figshare.com/account/items/23055962/edit**).**

## List of abbreviations

AOR: Adjusted odds ratio
ANC: Antenatal care
COR: Crude odds ratio
EDHS: Ethiopian demographic health survey
IFA: Iron folic acid
SPSS: Statistical package for social sciences
WHO: World health organization
